# Estimated incidence and Prevalence of noma in north central Nigeria, 2010–2018: A retrospective study

**DOI:** 10.1371/journal.pntd.0007574

**Published:** 2019-07-22

**Authors:** Seidu A. Bello, John A. Adeoye, Ifeoluwa Oketade, Oladimeji A. Akadiri

**Affiliations:** 1 Research Division, Cleft and Facial Deformity Foundation (CFDF), International Craniofacial Academy, Gwarinpa, Abuja, Federal Capital Territory, Nigeria; 2 Department of Oral and Maxillofacial Surgery, University of Port Harcourt Teaching Hospital, Rivers State, Nigeria; Hospital Infantil de Mexico Federico Gomez, UNITED STATES

## Abstract

**Background:**

Noma is a spreading and fulminant disease believed to be native to Sub-Saharan Africa over the last decade and associated with low socioeconomic status of citizens of the region. Within this noma belt, most epidemiological reports regarding the disease have emanated from the north western region of Nigeria. However, our indigenous surgical mission encountered a substantial number of cases of noma and post-noma defects noteworthy of epidemiological representation across north central Nigeria.

**Methods:**

All noma cases encountered within the 8-year study period were included and divided based on clinical signs into acute and sequelae groups. Incidence estimation was based on acute/recently active cases and was calculated using the statistical method proposed by the WHO Oral Health Unit (1994). Period prevalence of noma was calculated considering the population at risk in the zone.

**Findings:**

A total of 78 subjects were included in the study with age ranging from 2–75 years. Twelve subjects (15.4%) presented with acute disease while 66 (84.6%) had various forms of post-noma defects. The estimated incidence of noma in the north central zone was 8.3 per 100000 with a range of 4.1–17.9 per 100000 across various states. Period prevalence of noma which incorporated all cases seen within the study period was 1.6 per 100000 population at risk.

**Conclusion:**

Although noma may be more prevalent in the north western region of Nigeria, substantial number of cases occurs within the north central zone which calls for deliberate public awareness campaign on disease risk factors and prevention, and education of primary health-care providers.

## Author summary

Noma, a devouring disease of the orofacial complex, is commonly associated with poverty and impoverished regions of the world especially Sub-Saharan Africa termed the noma belt region of the world. With more reports advocating for full inclusion of noma in the WHO Neglected Tropical Diseases (NTDs) program, the apparent neglect of the disease in north central Nigeria compared to other sub-regions is worrisome as the disease burden in the sub-region has not been reported till date. In this light, a retrospective, cross-sectional survey was conducted to provide epidemiological representation to the cases encountered within an eight-year period by the Cleft and Facial Deformity Foundation (CFDF), an indigenous surgical mission. The incidence of noma was estimated using methods recommended by WHO while the period prevalence was calculated considering the population living below poverty line in the sub-region. This study extrapolates an incidence of 8.3 cases per 100000 and a period prevalence of 1.6 per 100000 persons at risk. Notable is the finding that most individuals encountered were above thirty years of age and suffered varying degree of facial disfigurement consequent to acute noma disease experienced in their childhood/early adolescence. Therefore, we advocate public awareness on the disease risk factors and prevention within the sub-region as well as training of primary health personnel on disease identification, primary care and nearest referral centres. We also identify the need to bolster the efforts of existing health facilities and indigenous surgical missions in the management and rehabilitation of noma cases and survivors.

## Introduction

Noma is a disease of the orofacial region that has been unanimously described as devastating, mutilating, destructive and debilitating due to its appearance and the nature of spread of the acute necrotizing lesion which runs fulminating courses. Alternatively known as Stomatitis gangrenosa or Cancrum oris, the aetiology of noma is infectious, yet unclear as regards the exact causative microorganism(s) [1,2]. Initially, a fuso-spirochetal microbial complex was implicated due to the higher level of these organisms in individuals with noma; however, this notion has been dispelled due to nonreproducibility in animal models following inoculation with these microorganisms under similar noma predisposing conditions [3]. Nonetheless, more recent breakthrough into the inquiry of noma microbiology has revealed a polymicrobial interaction between intraoral commensal organisms and extraoral opportunistic microbes as the most likely cause of the disease [2]. Although noma is almost exclusive to young children within ages two to six years; it has been shown to affect individuals across all age groups, progressing through unique clinical stages beginning at the reversible and seemingly inconsequential necrotizing gingivitis/oedema stages, to the grotesque gangrenous stage associated with extensive soft and hard tissue necrosis and a high mortality rate of 90% in untreated individuals [4–5]. In the presence of appropriate medical intervention at the latter stage of acute disease, scarring occurs–leaving sufferers with various forms of socially incapacitating facial defects which defines the chronic phase of the disease [5]. Despite being a disease first described over four centuries ago as affecting several world regions, noma is currently regarded as being exclusive to the tropics (notably sub-Saharan Africa) [6–7], which is accredited to the preponderance of noma predisposing factors in the region. These factors include socioeconomic factors such as low standards of living, extreme poverty, poor sanitary conditions and close proximity of residence to livestock. Oral conditions such as poor oral hygiene and presence of simple gingivitis; systemic conditions like severe malnutrition, measles, malaria, tuberculosis, HIV infection, leukaemia, Non-Hodgkin’s lymphoma and cyclic neutropenia; and miscellaneous factors including low birth weight, improper weaning, birth position within the family and absence of mother as primary care giver [4,6,8–9].

Epidemiological research targeted at determining noma incidence and prevalence has been highlighted as a main feature of public health action programs against the disease. As determination of actual epidemiological parameters of noma is difficult due to high mortality associated with untreated disease, regional health data record inadequacies, remoteness of affected areas in addition to sufferers’ lack of access to primary health centres; current epidemiological data estimates a global incidence of 30,000–40,000 cases annually with seventy-five percent of these occurring in sub-Saharan Africa (the noma belt) [9–10]. Furthermore, in Nigeria (particularly the north west and south west sub-regions), incidence rates between 0.8–6.4 per 1000 children have been reported in the last two decades mostly according to data provided by foreign non-governmental organizations or surgical missions [10–11].

Since 2010, our indigenous surgical mission (Cleft and Facial Deformity Foundation [CFDF]) has embarked on organizing free intervention programs for individuals with orofacial conditions and deformities requiring urgent or elective surgical intervention in north central Nigeria–a region challenged with the dearth of craniofacial surgical expertise in secondary and tertiary health institutions. Although it is widely presupposed that the noma scourge is exclusive to northwest Nigeria as evidenced by the number of reports that have emanated from the region and the establishment of an health institution–Noma Children Hospital, solely concerned with treatment of acute stages of the disease and rehabilitation of survivors in the sub-region; cases of noma have also been encountered and successfully managed by our surgical mission across north central Nigeria within eight years. As epidemiological data is important for planning and prioritisation of service delivery as well as formulation of disease preventive strategies, we aim to provide an epidemiological report on noma disease in north central Nigeria by determining the incidence, prevalence, trend and risk factors for noma in the sub-region based on the health data records of noma cases encountered by our foundation over an eight years period spanning from 2010 to 2018.

## Materials and methods

This is a retrospective cross-sectional, epidemiologic study involving the health and treatment records of all noma cases encountered at our various surgical outreach locations in north central Nigeria between 2010 and 2018. Cleft and Facial Deformity Foundation (CFDF) is an indigenous surgical nongovernmental organization that has its focus on providing free surgical care for individuals with orofacial diseases. The organization is based in the north central geo-political zone of Nigeria, with its Head office in the more central Federal Capital Territory, from where surgical missions are being embarked upon to different locations within the zone.

### Data collection

Comprehensive information was obtained from stored records of patients encountered in all surgical outreach programmes organized in north central Nigeria from June 2010 till September 2018. All cases diagnosed as noma were included in this study, and this comprised both individuals with the acute disease or its sequelae. Since noma and orofacial cleft may share some similarities in clinical presentation, cases of the latter were excluded based on their congenital nature of occurrence and absence of significant morbidity associated with the deformity. Other head and neck or orofacial disease conditions were also excluded.

The information obtained from the records included participants’ bio-data, year of encounter and facial location of defects at presentation. Distance between patients’ location of residence and the health institutions where the surgical outreaches were conducted was estimated for each participant. Other information included the number of siblings in the family (<18 years), proximity of residence to livestock (cattle, pigs, horses etc), primary caregiver around the time of disease onset, and history of visits to referral centres. Cases were also categorized into one of the five stages of the disease proposed by World Health Organization (WHO)–necrotizing gingivitis/beginner, oedema, gangrene, scar and sequelae; with the latter two stages indicative of long-standing or resolving disease [5].

In the custom of the Cleft and Facial Deformity Foundation Data Management Team (CFDF-DMT), all health records are scrutinized at the end of every outreach program and variables perceived to be missing from a patient’s record are identified and eventually obtained from them at recall visits (usually organized about two months following the surgical outreach program). For the purpose of this study, participants whose missing information could not be updated at the follow-up visits were excluded. This was necessary to ensure validity and reduce uncertainty of the research outcome.

### Statistical analysis

Data obtained from the study was analyzed using Statistical Package for Social Sciences (SPSS) version 23.0 (IBM Corp Armonk, NY, USA). Descriptive statistics such as frequencies, mean and standard deviation were explored for quantitative and categorical variables as appropriate. The normality of the distribution was ascertained using the Shapiro-Wilk’s test. Difference between quantitative variables was determined using the Mann-Whitney U test while relationships between categorical variables were determined using the Pearson’s Chi-square test. The significance value of all statistical tests used were set to 5% (p<0.05).

The prevalence of noma in the region was calculated by utilizing the total number of noma cases (both acute and chronic phase) seen within the study period as numerator and the population at risk as denominator. The population at risk considered only 45.7% of individuals residing below poverty line in the north central zone of Nigeria [12].

Incidence estimation analysis was done in line with the 1994 consultation report of the WHO Oral Health Unit using the Delphi method [13]. Only confirmed cases of acute noma (≤10years; beginner, oedema and gangrene) or older sequelae cases who marked their sixth birthday within the study period (giving due consideration to their year of encounter) were included in the analysis. According to the method, estimating the total incidence (I) involves a two-step process, beginning with the determination of the total surviving cases. The number of surviving cases (S) is expressed as a function of the number of referred cases reaching our outreach or resident centres (R) and an approximation of the percentage of the total surviving cases that were referred (χ) which was approximated as 15%, considering the notion that about ‘one out of every five’ noma cases presenting to referral centres [13] and the fact that our programs were carried out quarterly.

$$S=\frac{R\times 100}{\chi }$$

Thereafter, the total incidence (I) was extrapolated based on ‘S’ and the case survival rate of noma (y; 10%).

$$I=\frac{S\times 100}{y}$$

### Ethics

Ethical approval for the study was obtained from the Research Ethics review board of the International Craniofacial Academy. Prior notification of all outreach participants regarding the use of their health records and/or photographs for research purposes was done at each outreach event, with consents obtained. Records or images of participants/beneficiaries who refuse consent were never selected included or illustrated.

## Results

Within the eight-year study period, our indigenous surgical mission encountered 78 noma cases in twelve secondary health centres across Kogi, Nasarawa, Niger and the Federal Capital Territory of north central Nigeria; although, some centres in Kogi, Nasarawa and Niger states were visited at least twice between 2010 and 2018. A total of 12 subjects had acute noma while 66 participants presented with defects consistent with noma sequelae.

### Socio-demographic characteristics of subjects

Age and sex variables of the noma cases seen were not normally distributed (Shapiro-Wilk’s test, p<0.05). Participants encountered were within ages 2–75 years with a majority of 43.6% (n = 34) being above 30 years (Table 1). The average age of participants in this study was 29.6±18.84 years. Most individuals presenting with acute noma were between ages 2–10 years (n = 10; 83.3%), with two subjects being adults aged 30 and 35 years; while approximately half of the sequelae cases which accounted for the most of the cases seen were above 30 years of age (n = 33, 50%) [p = 0.001]. Analysis of their sex distribution revealed that males (n = 42, 53.8%) were slightly more than females (n = 36, 46.2%), with a similar pattern of distribution obtained for both acute and sequelae cases [p = 0.735]. Most noma cases were observed in centres within Niger and Nasarawa states (n = 48; 61.1%) with only nine cases (11.5%) recorded in Kogi state (Table 1). Four acute noma cases were encountered in Niger and Nasarawa states respectively (denoting most of the cases), while subjects with noma sequelae defects were mostly encountered in Niger state (n = 21; 31.8%). Since acute noma participants were mostly pre-schoolers or middle-age children, records based on occupation was only made to reflect if they were properly enrolled in school or not, which all participants in this category had no formal education at the time of encounter (Table 1). Comparatively in terms of economic status, most participants with noma sequelae (n = 28, 42.4%) were not productively engaged, while 18.2% had careers centred on agriculture (farming, fishing or cattle rearing).

**Table 1:** Socio-demographic distribution of all 78 noma cases encountered between 2010 and 2018.

		Total cases (%)	Active disease (%)	Sequelae (%)	Mean (SD)	p value
Age (in years)	<5	6 (7.7)	6 (50.0)		2.7 (1.03)	U = 74.500; P = 0.001*
	5–10	7 (9.0)	4 (33.3)	3 (4.5)	8.4 (1.72)	
	11–15	11 (14.1)		11(16.7)	13.5 (1.13)	
	16–20	7 (9.0)		7 (10.6)	17.7 (1.50)	
	21–25	7 (9.0)		7 (10.6)	23.9 (1.46)	
	26–30	6 (7.7)	1 (8.3)	5 (7.6)	28.6 (1.21)	
	>30	34 (43.6)	1(8.3)	33(50.0)	47.5 (12.55)	
Sex	Male	42 (53.8)	7 (58.3)	35 (53.0)		χ^2^ = 0.115; df = 1; p = 0.735
	Female	36 (46.2)	5 (41.7)	31 (47.0)		
Occupation	Child (uneducated)	13 (16.7)	10 (83.3)	3 (4.6)		
	Adolescent (uneducated)	7 (9.0)		7 (10.6)		
	Students	6 (7.7)		6 (9.1)		
	Farmers/Fishermen/Cattle rearers	12 (15.4)		12 (18.2)		
	Artisans	11 (14.1)	1(8.3)	10 (15.2)		
	Unemployed	29 (37.2)	1(8.3)	28 (42.4)		
Residential State	Federal Capital Territory (FCT)	21 (26.9)	3 (25.0)	18 (27.3)		χ^2^ = 0.224; df = 3; p = 0.974
	Kogi	9 (11.5)	1 (8.3)	8 (12.1)		
	Nasarawa	23 (29.5)	4 (33.3)	19 (28.8)		
	Niger	25 (32.1)	4 (33.3)	21 (31.8)		

*Statistically significant difference; P<0.05; Mann-Whitney U test

### Clinical stage, pattern and annual trend

Of the twelve (12) acute noma cases recorded, 91.7% exhibited features of gangrenous stage of the disease (n = 11), while only one subject was noted to have presented with facial swelling and necrotic ulcerations of the mucosal lining of the upper lip and cheek which are consistent with the oedema stage of noma. Thirteen of the 66 participants with post-noma defects (19.7%) had nascent scarring indicative of recent active disease, with 53 (80.3%) showing clinical signs that are indicative of stage five (full blown sequelae) of the noma disease spectrum. Regarding the facial locations of the noma defect, 50% of all cases (n = 39) had deformities involving the nose while 28.2% (n = 22) and 32.1% (n = 25) had lesions that affected their right and left cheek respectively. Of both lips, the upper lip was mostly affected (n = 30, 38.5% > n = 13, 16.7%), and the occurrence of trismus among noma sequelae sufferers was 13.6% (n = 9).

**Fig 1:**
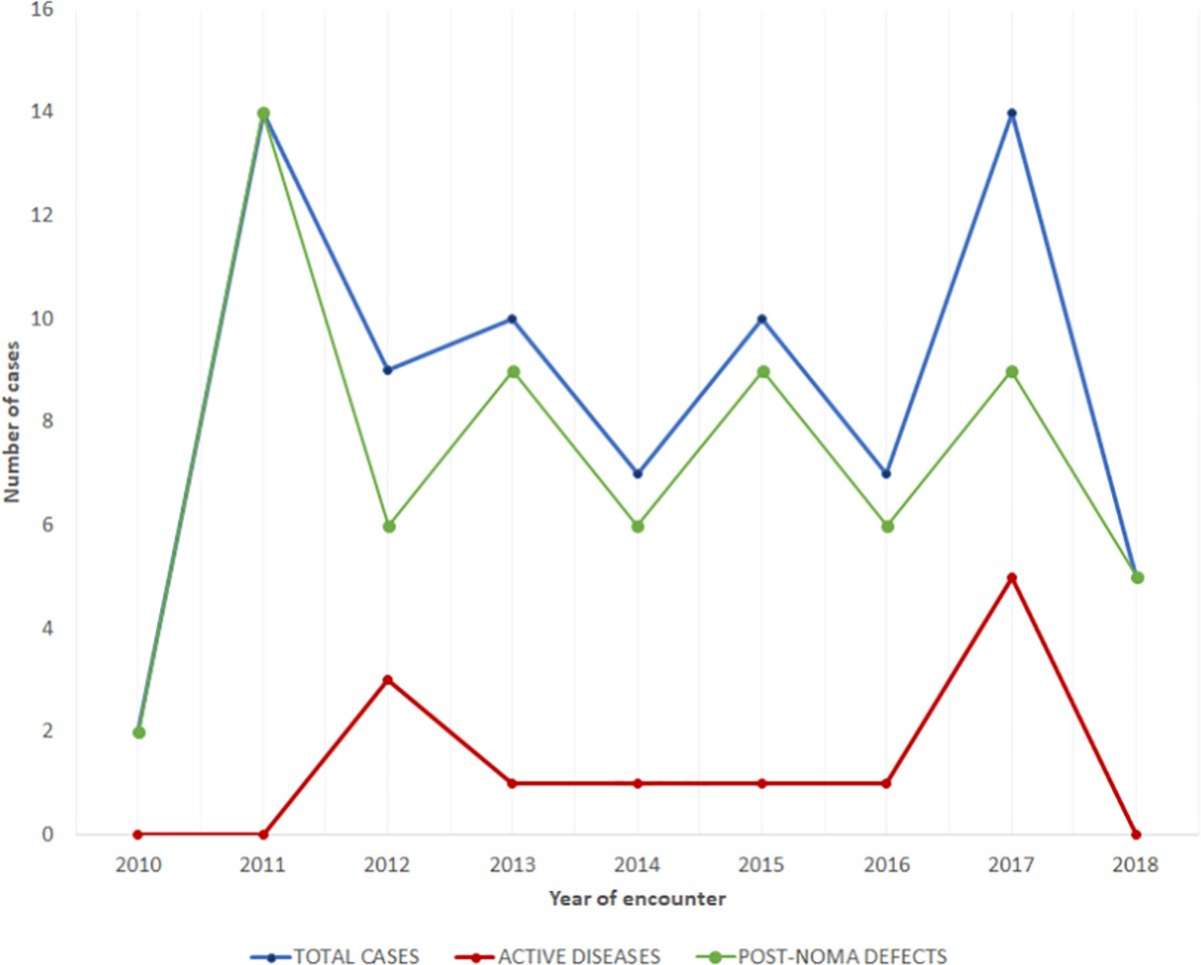
Annual trend of noma in north central Nigeria from 2010–2018.

Within the study period, acute noma cases were first encountered in 2012, with a steady rate of occurrence from 2013 to 2016, and increase in the number of cases encountered in 2017 (n = 5, 41.7%). However, no cases of acute noma were seen within the study period of 2018 (Fig 1). Participants with post-noma defects were seen annually throughout the study period, with cases increasing from two (3.0%) to fourteen (21.2%) from 2010 to 2011. Alternating decline and increase of noma sequelae cases were thereafter observed from 2011 to 2018 (Fig 1). The highest number of noma cases seen in a single year was 14 (17.9%), which occurred in 2011 and 2017. While all the participants encountered in 2011 had post-noma defects, five out of the fourteen cases in 2017 had acute noma lesions (35.7%). Table 2 shows the assortment of all noma participants (acute and chronic) presenting to the outreach referral centres within different parts of the zone.

**Table 2:** Distribution of all 78 noma cases seen in north central Nigeria (2010–2018) according to the year and centre of encounter.

	Centre of encounter			
	Federal Capital Territory (%)	Kogi (%)	Nasarawa (%)	Niger (%)
Year	2010	2 (9.5)			
	2011	6 (28.5)			8 (32.0)
	2012	9 (42.9)			
	2013	4 (19.1)		6 (26.1)	
	2014		2 (22.2)	5 (21.7)	
	2015		6 (66.7)	4 (17.5)	
	2016		1(11.1)	3 (13.0)	3 (12.0)
	2017			3 (13.0)	11 (44.0)
	2018			2 (8.7)	3 (12.0)

### Risk factor assessment for noma in north central Nigeria

Pertinent information collected regarding risk factors associated with noma included the number of siblings in the family, being raised by extended family members (especially grandparents), proximity of household residence to livestock and distance between residence and location of health outreach facility. The number of siblings of participants (< 18 years) ranged from 3 to 18 in total with an average of 8.6 ± 5.06. Furthermore, 85.9% (n = 67) answered positively that they lived in close proximity to livestock or even reared them while only 19.2% (n = 15) admitted to residing with extended relatives around the time of onset of noma disease.

The mean distance between patients’ residence and location of the health facility used for the surgical outreach was 124.8 ± 96.713km, with 41(52.6%) participants residing at locations 30 to 100 km from the referral centres. Twenty-eight (35.9%) individuals live between 101 to 300km away from the host secondary health facilities while 5 (6.4%) and 4(5.1%) participants dwell in areas <30km and >300 km from the centres respectively. Participants’ records further revealed that 60 (76.9%) subjects had never visited a health referral centre in the past and cited our indigenous surgical mission as the first centre of presentation since the disease onset.

### Estimated incidence of noma

The total estimated incidence of noma in the north central region of Nigeria between 2010 and 2018 is 8.3 per 100,000 population, with approximately 109 new cases diagnosed annually (17–42 cases across the various captured states, within the geopolitical zone). Noma incidence was highest in Nasarawa state with a rate of 17.9 cases per 100,000 population and lowest in Kogi state with an incidence of 4.1 cases per 100,000 population. The incidence extrapolated for Niger state and the Federal capital territory (FCT) was 5.1 and 14.2 per 100,000 respectively.

The period prevalence of noma in this study is 1.6 cases per 100,000 population at risk, with calculated sex occurrence rates being 1.7 per 100000 for males and 1.5 per 100000 for females. Noma prevalence was highest in the Federal capital territory (3.3 per 100,000 population at risk), with proportions ranging between 0.6–1.4 per 100,000 obtained in Kogi (0.6), Nasarawa (1.3) and Niger (1.4) states.

## Discussion

As a means of overcoming the challenges posed by the paucity of noma epidemiological data, nongovernmental organizations were one of relevant stakeholders saddled with the responsibility of reporting cases of noma sufferers and survivors encountered, in a bit to raise awareness on disease occurrence across their various stations or referral centres within the noma belt region [14]. Over the study period, our volunteer-based surgical mission discovered a substantial number of noma cases noteworthy of epidemiologic representation in north central Nigeria, which would allow for adequate characterization of the disease burden in this region–an area resident to the third-highest number of citizens living below poverty line in Nigeria [12]. In addition, our study shifts major attention from the recent norm of conducting noma epidemiological surveys in the north western region of Nigeria (Sokoto state in particular) over the last decade to the north central region comprising six member states (Benue, Kogi, Kwara, Nasarawa, Niger, Plateau) and the Federal Capital Territory.

Attempts at estimating noma incidence commenced towards the end of the 20^th^ century. Barmes et al [15] first reported case incidence extrapolations of noma from Niger, Nigeria and Senegal, which followed in 1998 with the world health report by Bourgeois and Leclerq–initially estimating an incidence of 140,000 cases worldwide from interviews with health workers in noma prevalent areas [16]. Fieger et al [10] from the most recent report on noma incidence in north west Nigeria, estimated an incidence of 25,600 cases in developing countries bordering the Sahara Desert (the noma belt of the world), and a global incidence of 30,000–40,000 cases. In like manner, our study estimates a noma incidence of 8.3 per 100000 in north central Nigeria from 2010–2018, with a range of 4.1–17.9 per 100000 observed across different states within the geopolitical zone. This estimate is approximately eighty folds less than the calculated incidence of noma reported by Fieger et al [10] from 378 noma patients encountered between 1996–2001 in Sokoto, Nigeria. In the latter study (based on a multiple logistic regression model of deducing unknown noma incidence from available incidence data of orofacial cleft within the region), the estimated incidence was 6.4 per 1000 with values varying between 4.4 and 8.5 per 1000 in individuals aged 10–30 years. Our lower incidence estimate may be clearly attributed to the wide variation in poverty indices of the north western and north central region of the country over several years, with the north western region serially recording the highest number of individuals living below poverty line in the country (>80.0%) [12]. By inference therefore, the north central zone may have less residents with severe malnutrition, unsafe drinking water, poorer sanitation practices and limited access to proper healthcare when compared to the north western sub-region. A supporting reason for the wide variation observed as compared to the reports of Fieger et al [10] may be the inclusion of noma cases above 10 years of age and possible noma sequelae cases in the sample utilized for the incidence estimation in the latter which implies probable over-estimation in the incidence values extrapolated for the north west region of Nigeria. Our calculated incidence was also lower than the values obtained by Denloye et al [17] in Ibadan, south west Nigeria, where an incidence rate of 7.0 per 1000 cases was reported in individuals within ages 1 to 12 years from 1986–2000. This finding may be attributed to the disparate methodology of incidence calculation in both studies, as the forty-five noma cases reviewed by Denloye et al [17] were used against the total number of children that presented to the referral centre within the study period.

In further comparison of our findings with previous reports from other countries within the noma belt of the world, our estimated incidence was lower than the case incidence reported from Niger Republic (1.34 per 1000) and Senegal (0.7–1.2 per 1000) by Barmes et al [15] among children aged 0–6 years. However, this comparison may be flawed since at the time of data extrapolation, a seemly unrealistic mortality rate of 70% was utilized for the incidence estimation in their study; this observation was also highlighted by the reports of Fieger et al [10]. Subsequently, incidence estimates adjusted to a more accurate noma mortality rate of 90% resulted in incidence estimates between 1.2 to 4.2 cases per million in Dakar, Senegal among children aged 0–9 years of age [18], which ranks lower than the calculated incidence in our study.

The geographic distribution of noma is commonly represented figuratively on world maps by the WHO and its Regional offices, and titled “Noma in the world” [13]. These maps, which were first published in 1994, were made to depict reported cases of the disease across various parts of the world based on available data at the time of publication; hence, providing a diagrammatic panorama of the current noma situation in the world. The last update of these maps (published around 2009) [19] showed then recent noma case observations in 67.9% of African countries, no recent reported cases in two countries (DR Congo and Morocco) and sixteen “noma free” countries (Swaziland, Lesotho, Liberia, Sierra Leone, Guinea Bissau, Congo, Gabon, Libya, Tunisia, Equatorial Guinea, Burundi, Rwanda, Eritrea, Comoros, Mauritius and Western Sahara) within the African continent. Other affected continents included Asia (India, Pakistan, Myanmar), South America (Colombia, Guyana, Suriname, Argentina, Paraguay and Uruguay), with sporadic reports in Oceania, Europe and North America. With no recent map publication for over a decade, there is no current ‘snapshot’ or precise description of noma case observations worldwide, as well as no performance indicators of preventive strategies targeted at disease prevalent areas. Therefore, an update of these ‘noma maps’ by relevant monitoring stakeholders based on recent data/reports from experts within the last decade is urgently required in Africa and indeed globally to allow for knowledge acquisition on current ‘high risk’ areas and concomitant implementation of primary and secondary preventive approaches in these regions.

The pattern of noma/post-noma soft tissue defects in north central Nigeria includes varying degree of deformities involving the nose, upper lip, left cheek, right cheek and lower lip in decreasing order of occurrence. Our finding corroborates the pattern of noma presentation observed in Dakar Senegal in which the upper lip then cheek were quoted as the two most common sites affected by the noma defect; although, cases involving the nose were not cited in their report [18]. In contrast, the site distribution of defects in our study varies from the recent observations of Adeniyi and Awosan in Sokoto, north west Nigeria where lesions involving the cheeks were mostly seen, followed by upper and lower lips with the nose being the least affected site of soft tissue defects [20].

Farley et al [9] in a case-control study involving 74 cases and 222 controls in north west Nigeria, associated “caretaker” (i.e third party carer) as a factor that may influence the risk of developing noma. This was supported by Adeola et al [21] as reported in a case series involving five subjects managed for acute phase of noma in north west Nigeria. In that study, they asserted there was increasing number of noma cases in the region associated with lack of direct maternal care after children were weaned. This observation was not common in our study where only 19.2% of noma patients were being cared for by extended relatives around the time of disease onset. Although this proportion may be considerable, it was not significant enough to corroborate the observation of the earlier authors. Hence, it may require further studies to determine the plausibility of this assertion. Another important observation is the travel distance to access health care for patients in the north central sub-region. In this study, subjects had to travel about 125km on the average to access care from our treatment teams at the secondary health centres. In fact, 76.9% of them had not presented to any referral centre previously during the course of active disease or after its remission. Our experience in the region attributes this observation to factors such as subject’s preference for self-medication or traditional medical alternatives, lack of primary/secondary health centres within close proximity to residence, presence of primary centres but lack of required expertise and facilities for treatment, available facilities and expertise within state of residence but lack of finances to offset the cost of treatment and additional cost of transportation.

Since the extrapolations in this study were based on data records obtained from four states within the sub-region, the non-availability of records from Plateau, Benue and Kwara states represents a limitation to our incidence estimation; although, most areas are largely sub-urban/urban with tertiary health institutions in each of the states mentioned. Also, the possibility that some of the subjects may have developed the disease in these neighbouring north central states previously and relocated to the state of encounter prior to the outreach cannot be totally ruled out. Another limitation is the inability to explore in details, some major risk factors for noma due to the retrospective nature of the study design. Furthermore, the use of non-probability (convenience) sampling methods to arrive at the sample size employed for the analysis of associated risk factors for noma in this study may have introduced selection bias.

Considering that our study sought out to primarily determine the estimated incidence of noma in north central Nigeria, the results that have emanated are specific to characterizing the burden of noma in this zone. However, the statistical methods for the calculations done were adapted from the WHO Oral Health Unit and could as well be applied to other regions of the country or in other locations within the noma belt.

## Conclusions and recommendations

The estimated incidence of noma in north central Nigeria is 8.3 cases per 100000 population. Although the noma scourge is deemed prevalent in north west Nigeria and Sokoto state in particular, substantial number of cases is being encountered in the north central zone. Hence, efforts should be intensified in terms of public awareness, establishment of new primary health centres in deficient councils/wards, and education of community health workers in existing primary health care centres on disease identification (possibly primary care) in order to facilitate presentation of sufferers to appropriate referral centres within the north central zone. With a prevalence of 1.6 per 100000 population at risk and majority living with post-noma defects, it is clear that attention to surgical rehabilitation in the region is also suboptimal. It is therefore imperative in the absence of any health facility solely dedicated to the management and rehabilitation of noma patients in the region (unlike the northwest); that existing secondary health centres and nongovernmental organizations in the zone be better equipped to mitigate the disease burden and provide standard care for noma cases and survivors, especially as the poverty index of the zone and country is increasing. We further recommend that maps denoting noma occurrence in Africa and globally be updated according to recent available data so as to reflect current disease distribution and enable targeted preventive strategies in identified ‘high risk’ nations.

## Supporting information

S1 Checklist STROBE checklist. (DOC)
